# Effect of cariprazine on attention and quality of life in patients with predominant negative symptoms of schizophrenia: A post-hoc analysis

**DOI:** 10.1016/j.scog.2025.100355

**Published:** 2025-03-08

**Authors:** Oloruntoba J. Oluboka, Andrea Bardell, Howard C. Margolese, Philip G. Tibbo, Lisa Buchy, Christine Di Cresce, Jun Yu, Roger S. McIntyre

**Affiliations:** a Department of Psychiatry, University of Calgary, Calgary, AB, Canada; b The Ottawa Hospital Research Institute, The Ottawa Hospital, University of Ottawa, Ottawa, Canada; c University of British Columbia, Vancouver, Canada; d Department of Psychiatry, McGill University, Montreal, Canada; e Department of Psychiatry, Dalhousie University, Halifax, Canada; f AbbVie Corporation, Saint-Laurent, Canada; g AbbVie Corporation, Sugar land, USA; h Department of Psychiatry and Pharmacology, University of Toronto, Toronto, Canada

**Keywords:** Cariprazine, Negative symptoms, Schizophrenia, Attention

## Abstract

**Background:**

Cariprazine, a potent dopamine D_3_-preferring D_3_/D_2_ receptor partial agonist, has demonstrated benefits on negative symptoms among patients with schizophrenia. Secondary endpoint and post-hoc analyses have also suggested a benefit of cariprazine on quality of life (QoL) and attention.

**Methods:**

Data for this *post-hoc* analysis were pooled from two 6-week, placebo-controlled phase 3 trials evaluating cariprazine among patients with acute exacerbations of schizophrenia. One study included an aripiprazole active-control arm for assay sensitivity.

Two populations were analyzed: pooled intention-to-treat (ITT) population (*N* = 1043), and the pooled subgroup with predominant negative symptoms (PNS, *n* = 215), as defined by the Positive and Negative Syndrome Scale (PANSS) subscale and item cut-off criteria at baseline. Analyses of interest were: Schizophrenia Quality of Life Scale Revision 4 (SQLS-R4) total score; Cognitive Drug Research (CDR) power of attention (PoA), and continuity of attention (CoA).

**Results:**

Among study completers, cariprazine and aripiprazole were associated with significant SQLS-R4 improvements in the ITT and PNS populations. Differences in CDR-PoA scores were significant for cariprazine vs. placebo in the ITT and PNS populations, but not for aripiprazole in the ITT or PNS analyses. Differences in CDR-CoA scores were significant for cariprazine vs. placebo in the ITT and PNS analyses; and was significant for aripiprazole vs. placebo in the PNS analysis, but not in the ITT analysis.

**Conclusions:**

This post-hoc analysis suggests that cariprazine may be associated with beneficial effects on measures of attention and QoL among patients with schizophrenia, and these effects could be more pronounced among individuals with PNS.

## Highlights

• Cariprazine has previously demonstrated benefits on negative symptoms of schizophrenia.
• This post-hoc analysis included a subgroup of people with schizophrenia with predominant negative symptoms (PNS).
• Analysis showed that cariprazine may be associated with benefit on measures of attention and quality of life.
• Cariprazine's beneficial effects may be more pronounced among individuals with PNS.

## 1 Introduction

Individuals with schizophrenia experience other symptoms in addition to the hallmark positive symptoms of psychosis (e.g., delusional beliefs, hallucinatory experiences). Negative symptoms (e.g., avolition, restricted emotional expression) and cognitive impairment are often present, may be overlooked, and can persist in patients who have achieved stability of their positive symptoms. (Correll and Schooler, 2020; Suttajit and Pilakanta, 2015; Rabinowitz et al., 2013a, Rabinowitz et al., 2013b; Hunter and Barry, 2012; Milev et al., 2005).

Negative symptoms affect up to 60 % of individuals with schizophrenia (Bobes et al., 2010). Approximately 30 % experience primary negative symptoms (also known as deficit symptoms) that are sufficiently prominent to warrant clinical attention (Buchanan and Gold, 1996; Stahl and Buckley, 2007). These deficit symptoms are core features of the illness, and not secondary to positive symptoms, comorbid depression, medication side effects, substance abuse, or social isolation (Mosolov and Yaltonskaya, 2022). Up to 75 % of individuals with schizophrenia will also experience cognitive deficits (e.g., problems with executive function, processing speed, verbal and/or working memory) (Harvey, 2019; Solé et al., 2017; Raffard et al., 2009). The impact of both negative and cognitive symptoms can be substantial; including poor social and occupational functioning and a profound impact on daily life (Hunter and Barry, 2012; Milev et al., 2005). Particularly, cognitive deficits have been associated with increased frequency of hospitalizations, longer duration of illness, greater burden of positive and negative symptoms, and lower psychosocial functioning (Gkintoni et al., 2017; Kuswanto et al., 2016).

D_2_ receptor antagonism or partial agonism is a common characteristic of nearly all approved antipsychotic drugs. D_2_-receptor blockade is associated with improvement in positive symptoms of schizophrenia, but generally not improvement of negative symptoms or cognitive functioning (Correll and Schooler, 2020; Correll et al., 2020; Keefe et al., 2007). Currently, there is no pharmacotherapy specifically approved to treat either negative symptoms or cognitive impairment; this is recognized as an unmet medical need (Martinez-Aran and Vieta, 2015; Kirkpatrick et al., 2006).

The dopamine D_3_-receptor is postulated as an important target for treating negative and cognitive symptoms of schizophrenia (McIntyre et al., 2022; Calabrese et al., 2020; Sokoloff and Le Foll, 2017; Stahl, 2017; Maramai et al., 2016; Neill et al., 2016; Nakajima et al., 2013). With respect to cognition, evidence suggests that D_3_-receptor blockade may improve domains of memory, attention, learning, processing speed, social recognition, and executive function (Nakajima et al., 2013).

Cariprazine is a potent dopamine D_3_-preferring D_3_/D_2_receptor partial agonist and serotonin 5-HT_1A_ receptor partial agonist. It is approved as monotherapy for schizophrenia and bipolar I disorder and as an adjunctive therapy for major depressive disorder (AbbVie Corporation (Canada), 2022; AbbVie Inc. (USA), 2023). It has up to 8-fold greater affinity for dopamine D_3_ than D_2_ receptors in vitro and high in vivo occupancy of both D_3_ and D_2_ receptors (Girgis et al., 2016; Kiss et al., 2010; Kiss et al., 2012). Clinical trial evidence and post-hoc analyses suggest a particular benefit of cariprazine on negative symptoms, cognition and quality of life (QoL) (McIntyre et al., 2022; Németh et al., 2017; Durgam et al., 2015; Kane et al., 2015; Kahn et al., 2015).

In a randomized, 26-week, head-to-head, double-blind, phase 3b trial (Németh et al., 2017), patients with schizophrenia with predominant negative symptoms (PNS) treated with cariprazine demonstrated significantly greater improvement across all assessments of negative symptoms and social functioning than patients treated with risperidone. PNS was defined in the trial by clinical stability and predominant negative symptoms in the 6 months preceding the trial. Additional exclusion criteria were implemented to minimize the potential impact of secondary improvements in other psychopathological domains (i.e., pseudospecific factors) on observed negative symptoms outcomes. Note that while the study was designed to maximize inclusion of patients with primary negative symptoms, the “predominant” terminology was used in recognition of the possibility that some negative symptoms may have been due to secondary causes.

With respect to cognitive symptoms, studies of acute exacerbation of schizophrenia (Kane et al., 2015; Durgam et al., 2015) and *post-hoc* analyses (McIntyre et al., 2022) have suggested an improvement in specific domains (e.g., attention) with cariprazine compared to placebo. Statistically significant benefits were noted on measures of both focused and sustained attention using the Cognitive Drug Research (CDR) computerized, performance-based attention battery. The CDR consists of 3 brief and highly sensitive tests (simple reaction time, digit vigilance, and choice reaction time) organized into composite scores (Simpson et al., 1991). The two composites used in these analyses were power of attention (PoA: measuring focused attention) and continuity of attention (CoA: measuring sustained attention).

Further analyses have shown significant improvements in QoL with cariprazine vs. placebo using the Schizophrenia Quality of Life Scale Revision 4 (SQLS-R4) total score and vitality subscale score (Kane et al., 2015; Durgam et al., 2015; Kahn et al., 2015). The SQLS-4 is a disease-specific, subjective, self-administered questionnaire divided into two QoL sub-scales: cognition and vitality; and psychosocial feelings (Martin and Allan, 2007).

Given this background, the current *post-hoc* analysis was designed to provide more information about the benefits of cariprazine among patients with PNS, focusing on changes in cognition and QoL. The main objectives were to: (1) evaluate short-term changes on QoL and attention in schizophrenia patients treated with cariprazine versus placebo, and 2) further evaluate these changes within a subgroup of patients with PNS.

## 2 Methods

Subjects for this post-hoc analysis were pooled from two 6-week, phase 3 trials evaluating cariprazine in patients with acute exacerbations of schizophrenia (Durgam et al., 2015; Kane et al., 2015). Both studies were placebo-controlled and one study (Durgam et al., 2015) also included a 10 mg/d aripiprazole active-control reference arm.

In both studies, the primary efficacy outcome was the change in Positive and Negative Syndrome Scale (PANSS) total score from baseline to week 6. Additional efficacy measures included the changes from baseline in Clinical Global Impressions – Severity of Illness (CGI—S) score, measures of QoL (assessed with the SQLS-R4), and attention (assessed with the CDR-PoA and CDR-CoA).

For this analysis, two separate populations of subjects were analyzed: the pooled intent-to-treat (ITT) population of the two studies, and a subgroup of subjects from the ITT-population with PNS. PNS was defined for this analysis as a PANSS factor score for negative symptoms (PANSS-FSNS) of 24 or greater, a PANSS-Factor Score for Positive Symptoms (PANSS-FSPS) of 19 or lower; and scores of at least 4 on at least 2 of 3 specific PANSS items (N1: blunted affect; N4: social withdrawal; and N6: lack of spontaneity) (Earley et al., 2019). This definition identifies a subpopulation of trial patients with moderate-to-severe negative symptoms and minimal predominance of positive symptoms.

For each of the three main analyses, (SQLS-R4, CDR-PoA, and CDR-CoA), least-squares mean change (LSMC) from baseline to week 6 and least-squares mean difference (LSMD) of change for cariprazine vs. placebo and aripiprazole vs. placebo were calculated for ITT and PNS populations. PANSS total score, PANSS-FSNS, PANSS-FSPS and CGI-S score were also assessed. Pearson correlation coefficients were calculated to examine relationships between each pair of PANSS total, PANSS-FSNS, CDR-POA, and SQLS-R4 scores at baseline and changes after 6 weeks of treatment in both the ITT and PNS populations. Additionally, the rate of response (≥20 % reduction in PANSS total score or subscale scores) was calculated for each population at each week from baseline to week 6. Absolute response rates at week 6 were used in a number-needed-to-treat calculation.

Statistical analyses for both ITT and PNS populations were performed using two separate data sets: completer analysis (among individuals who had data available for baseline through week 6) and last visit analysis (using data from baseline up to the last data collected before discontinuation or completion). This report primarily focuses on the completer analysis, as studies of antipsychotics have shown that magnitude of treatment effects, particularly with respect to QoL, increase with duration of treatment (e.g., Bobes et al., 1998; Hertling et al., 2003; Strakowski et al., 2005). The results of the last visit analysis are also referred to below and are presented in detail in the supplementary data file.

Safety and tolerability of cariprazine among patients with schizophrenia have been well documented in the clinical trial program and were not assessed in this post-hoc analysis (Durgam et al., 2015; Kane et al., 2015).

## 3 Results

The pooled ITT population consisted of 1043 subjects, including 294 in the placebo group, 599 in the cariprazine group and 150 in the aripiprazole group. The PNS subgroup consisted of 215 subjects, including 66 in the placebo group (22.4 % of placebo patients), 105 in the cariprazine group (17.5 % of cariprazine patients) and 44 in the aripiprazole group (29.3 % of aripiprazole patients). The proportions of each group who completed the studies ranged from 62.2 % to 76.0 % across groups in the ITT population, and from 63.6 % to 77.3 % in the PNS subgroups. Baseline characteristics were found to be broadly similar across the groups (Table 1).

**Table 1:** Baseline characteristics.

	ITT (*n* = 1043)			PNS (*n* = 215)		
	CAR (*n* = 599)	PBO (*n* = 294)	ARI (*n* = 150)	CAR (*n* = 105)	PBO (*n* = 66)	ARI (*n* = 44)
Sex, n (%)						
Female	180 (30.1)	93 (31.6)	57 (38.0)	39 (37.1)	27 (40.9)	14 (31.8)
Male	419 (69.9)	201 (68.4)	93 (62.0)	66 (62.9)	39 (59.1)	30 (68.2)
Race, n (%)^a^						
Asian	111 (19.6)	56 (19.9)	2 (1.5)	15 (14.6)	12 (19.0)	2 (4.9)
Black	171 (30.2)	90 (31.9)	33 (24.6)	20 (19.4)	11 (17.5)	9 (22.0)
White	259 (45.7)	118 (41.8)	97 (72.4)	65 (63.1)	37 (58.7)	30 (73.2)
Other	26 (4.6)	18 (6.4)	2 (1.5)	3 (2.9)	3 (4.8)	0
Age, mean (SD), years	37.1 (10.3)	37.4 (11.3)	39.6 (10.7)	37 (10.8)	39.4 (11.9)	39.1 (10.5)
Weight, mean (SD), kg	75.5 (18.6)	75.8 (19.2)	79.6 (17.1)	73.9 (16.5)	75.4 (19.9)	77.3 (16.4)
BMI, mean (SD), kg/m^2^	25.8 (5.2)	26.1 (5.5)	26.9 (5.5)	25.2 (5.1)	26.5 (5.5)	25.7 (4.4)
Duration of illness, mean (SD), years	11.3 (9.1)	11.6 (9.7)	12.5 (8.9)	10.3 (7.8)	12.6 (9.9)	10.9 (7.6)
Baseline scores, mean (SD)						
PANSS Total	96.1 (9.1)	96.6 (9.2)	95.7 (9.0)	97.7 (8.0)	97.7 (8.4)	96.9 (7.9)
PANSS -FSNS	22.9 (4.5)	23.6 (4.5)	23.4 (4.4)	27.4 (2.8)	27.5 (3.0)	27.9 (3.1)
PANSS-FSPS	20.2 (3.2)	20.0 (3.4)	19.3 (3.3)	17.0 (2.0)	16.8 (1.9)	16.6 (1.7)
CGI-S	4.8 (0.7)	4.8 (0.7)	4.8 (0.6)	4.8 (0.5)	4.6 (0.6)	4.7 (0.5)
SQLS-R4	57.5 (21.1)	58.9 (22.2)	58.4 (21.8)	59.2 (20.5)	54.4 (20.0)	56.0 (22.7)
CDR-PoA	1972.3 (1165.7)	1997.4 (1124.9)	1812.6 (932.2)	2005.1 (1011.1)	2127.3 (1141.7)	2049.9 (1081.1)
CDR-CoA	79.8 (16.5)	79.9 (17.1)	82.8 (14.3)	78.6 (16.0)	78.8 (17.9)	76.8 (18.5)
Completed the study, n (%)	383 (63.9)	183 (62.2)	114 (76.0)	69 (65.7)	42 (63.6)	34 (77.3)

Abbreviations: ARI, aripiprazole; BMI, body mass index; CAR, cariprazine; CGI—S, Clinical Global Impressions – Severity of Illness; CDR-CoA, Cognitive Drug Research - continuity of attention; CDR-PoA, Cognitive Drug Research power of attention; ITT, intention to treat; PANSS, Positive and Negative Syndrome Scale; PANSS-FSNS, Positive and Negative Syndrome Scale factor score for negative symptoms; PANSS-FSPS, Positive and Negative Syndrome Scale factor score for positive symptoms; PBO, placebo; PNS, predominant negative symptoms; SD, standard deviation; SQLS-R4, Schizophrenia Quality of Life Scale Revision 4.

a Unspecified race: *n* = 32 for CAR (ITT), *n* = 16 for ARI, *n* = 12 for PBO (ITT), n = 2 for CAR (PNS), *n* = 3 for ARI (PNS) n = 3 for PBO (PNS).

### 3.1 Quality of life (SQLS-R4)

**Fig. 1:**
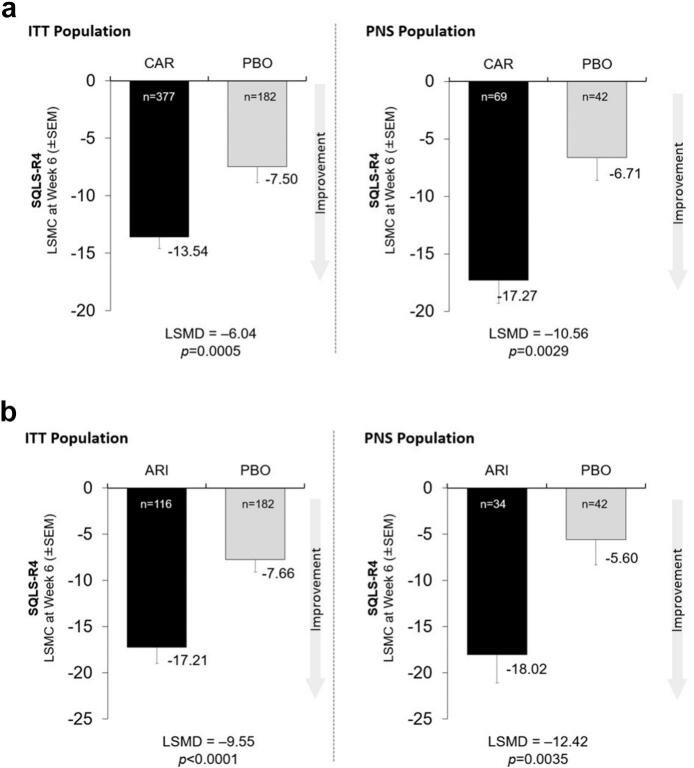
Least-square mean changes (LSMC) (± Standard Error of the Mean [SEM]) and least-square mean differences (LSMD) in the Schizophrenia Quality of Life Scale Revision 4 (SQLS-R4) score for cariprazine (CAR; Fig. 1A) or aripiprazole (ARI; Fig. 1B) vs. placebo (PBO) in completers from the intention-to-treat (ITT) and predominant negative symptom (PNS) populations at week 6.

Differences in SQLS-R4 scores for both cariprazine and aripiprazole vs. placebo were found to be statistically significant at week 6 for both the ITT and PNS populations. Among completers, the LSMD in favor of cariprazine was −6.04 for the ITT population (*p* = 0.0005) and − 10.56 for the PNS population (*p* = 0.0029; Fig. 1A). In the aripiprazole analysis, the LSMD in favor of active therapy was −9.55 for the ITT population (*p* < 0.0001) and − 12.42 for the PNS population (*p* = 0.0035; Fig. 1B). Using last-visit data, QoL improvements were significant for the ITT and PNS groups for both cariprazine and aripiprazole (Supplementary Figs. S1A and S1B).

### 3.2 Focused attention (CDR-PoA)

**Fig. 2:**
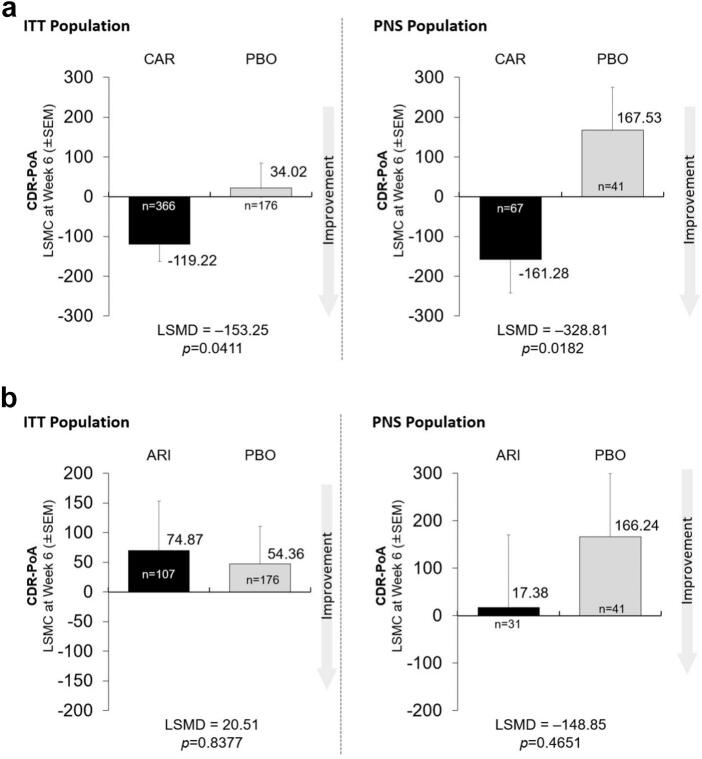
Least-square mean changes (LSMC) (± Standard Error of the Mean [SEM]) and least-square mean differences (LSMD) in focused attention based on the Cognitive Drug Research - Power of Attention (CDR-PoA) score for cariprazine (CAR; Fig. 2A) or aripiprazole (ARI; Fig. 2B) vs. placebo (PBO) in completers from the intention-to-treat (ITT) and predominant negative symptom (PNS) populations at week 6.

In the CDR-PoA analyses of cariprazine vs. placebo among completers, there were significant differences with cariprazine vs. placebo in the ITT population and in the PNS population (LSMD -153.25, *p* = 0.0411 and LSMD -328.81, *p* = 0.0182, respectively; Fig. 2A). For aripiprazole vs. placebo among completers, there was no significant difference shown in either of the ITT or PNS analyses (LSMD 20.51, *p* = 0.8377 and LSMD -148.85, *p* = 0.4651, respectively; Fig. 2B). Using last-visit data, there were no differences found among treatments for either the ITT or PNS populations (Supplementary Figs. S2A and S2B).

### 3.3 Sustained attention (CDR-CoA)

**Fig. 3:**
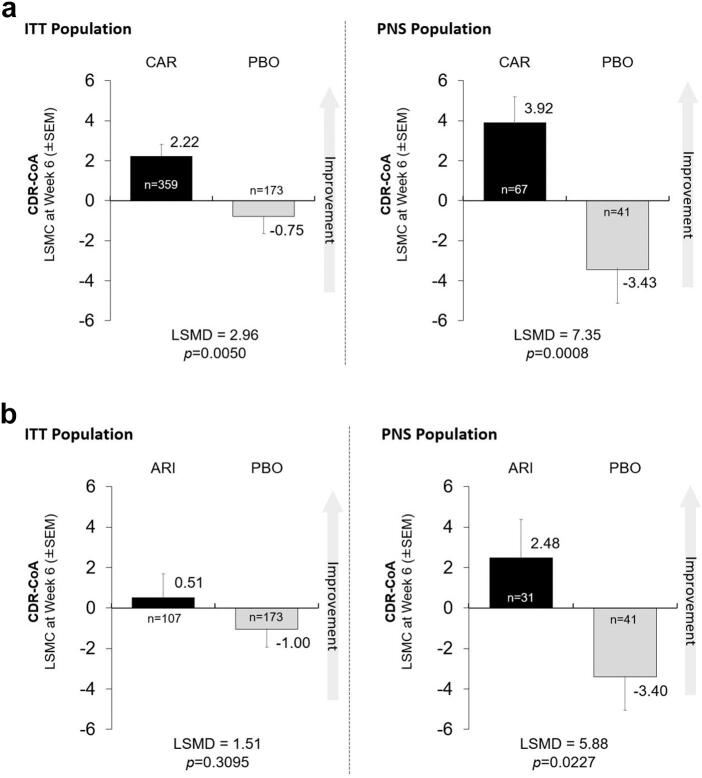
Least-square mean changes (LSMC) (± Standard Error of the Mean [SEM]) and least-square mean differences (LSMD) in Sustained Attention based on the Cognitive Drug Research - Continuity of Attention (CDR-CoA) score cariprazine (CAR; Fig. 3A) or aripiprazole (ARI; Fig. 3B) vs. placebo (PBO) in completers from the intention-to-treat (ITT) and predominant negative symptom (PNS) populations at week 6.

For the CDR-CoA analysis, there were significant improvements in favor of cariprazine vs. placebo in both the ITT and PNS populations (LSMD 2.96, *p* = 0.0050; and LSMD 7.35, *p* = 0.0008, respectively Fig. 3A). For aripiprazole vs. placebo, there was no significant difference in the ITT analysis (LSMD 1.51, *p* = 0.3095), but there was a significant difference shown in the PNS population (LSMD 5.88, *p* = 0.0227; Fig. 3B). In last-visit analyses, differences in favor of cariprazine were significant for both the ITT and PNS populations (Supplementary Fig. S3A). For aripiprazole, there were no differences shown in either population (Supplementary Fig. S3B).

### 3.4 PANSS and CGI-S scores (Table 2)

In the ITT analyses, PANSS total score, PANSS-FSNS, PANSS-FSPS and CGI-S scores were all improved significantly by each active therapy vs. placebo among completers. In the PNS population, significant benefit for cariprazine was found for the PANSS total score, PANSS-FSNS and PANS-FSPS. The between-group difference was not significant for the CGI-S analysis. For aripiprazole in the PNS analysis, there were no significant differences found for any of the metrics. The analyses using last visit data produced similar results (Supplementary Table S1).

**Table 2:** Changes in PANSS Total Score, PANSS-FSNS, PANSS-FSPS and CGI-S (Completers).

2A. Cariprazine vs. Placebo						
	ITT (*n* = 569)			PNS (*n* = 112)		
Score, LSMC (SEM)	CAR (*n* = 385)	PBO (*n* = 184)	LSMD	CAR (*n* = 69)	PBO (*n* = 43)	LSMD
PANSS Total Score	−26.35 (0.77)	−19.13 (1.1)	−7.22^a^	−27.80 (1.59)	−21.63 (2.02)	−6.17^b^
PANSS-FSNS	−5.35 (0.23)	−3.71 (0.33)	−1.63^a^	−7.58 (0.56)	−5.53 (0.70)	−2.04^c^
PANSS-FSPS	−7.11 (0.20)	−5.50 (0.29)	−1.61^a^	−6.06 (0.36)	−4.72 (0.46)	−1.35^d^
CGI-S	−1.61 (0.04)	−1.17 (0.06)	−0.44^a^	−1.54 (0.08)	−1.28 (0.11)	−0.25^e^
^a^*p* < 0.0001; ^b^*p* = 0.0180; ^c^*p* = 0.0247; ^d^*p* = 0.023; ^e^*p* = 0.0683						


2B. Aripiprazole vs. Placebo						
	ITT (*n* = 299)			PNS (*n* = 77)		
Score, LSMC (SEM)	ARI (*n* = 115)	PBO (n = 184)	LSMD	ARI (*n* = 34)	PBO (n = 43)	LSMD
PANSS Total Score	−24.90 (1.48)	−19.11 (1.17)	−5.79^a^	−25.97 (2.67)	−21.33 (2.37)	−4.63^c^
PANSS-FSNS	−5.05 (0.42)	−3.86 (0.33)	−1.19^b^	−6.39 (0.82)	−5.62 (0.73)	−0.76^d^
PANSS-FSPS	−6.57 (0.38)	−5.38 (0.30)	−1.18^b^	−5.68 (0.59)	−4.49 (0.53)	−1.19^e^
CGI-S	−1.59 (0.09)	−1.16 (0.07)	−0.44^a^	−1.51 (0.14)	−1.21 (0.12)	−0.30^f^
^a^*p* < 0.05; ^b^*p* < 0.01; ^c^*p* = 0.1981; ^d^*p* = 0.4907; ^e^*p* = 0.1384; ^f^*p* = 0.103						

Abbreviations: ARI, aripiprazole; CAR, cariprazine; CGI—S, Clinical Global Impressions – Severity of Illness; ITT, intention to treat; LSMC, least squares mean change; LSMD, least squares mean difference; PANSS, Positive and Negative Syndrome Scale; PANSS-FSNS, Positive and Negative Syndrome Scale factor score for negative symptoms; PANSS-FSPS, Positive and Negative Syndrome Scale factor score for positive symptoms; PBO, placebo; PNS, predominant negative symptoms.

**Fig. 4:**
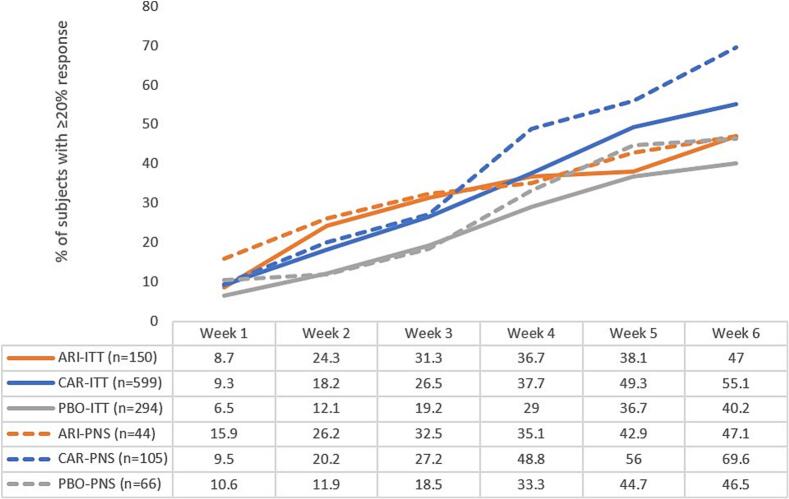
Percentage of subjects with ≥20 % response in the Positive and Negative Syndrome Scale factor score for negative symptoms (PANSS-FSNS) by week, from week 1 to 6 for aripiprazole (ARI), cariprazine (CAR), and placebo (PBO); in both intention-to-treat (ITT) and predominant negative symptom (PNS) populations.

Absolute and percent changes in PANSS scores over time were also investigated. The proportions of subjects who had a 20 % or greater PANSS-FSNS response are shown in Fig. 4, broken down by treatment and presence or absence of PNS. After 6 weeks, the PANSS-FSNS response rates in the ITT population were 40.2 % in the placebo group, 47.0 % in the aripiprazole group, and 55.1 % in the pooled cariprazine group. These rates in the PNS population were 46.5 %, 47.1 %, and 69.6 %; respectively. In a corresponding number-needed-to-treat (NNT) analysis, the NNT to achieve a 20 % or greater PANSS-FSNS response after 6 weeks in the ITT population was 15 for aripiprazole compared to placebo and 7 for cariprazine compared to placebo. For the PNS population, the NNT values for this outcome were 167 for aripiprazole compared to placebo and 5 for cariprazine compared to placebo.

### 3.5 Correlation analysis (Table 3)

Pearson correlation coefficients were calculated to evaluate associations between each pair of PANSS total, PANSS-FSNS, CDR-POA, and SQLS-R4 scores at baseline and changes after 6 weeks. As expected, there were significant correlations between PANSS total and PANSS-FSNS scores in all groups across all populations at baseline, and the change in these scores at week 6 (*p* ≤ 0.0001 in all instances). There was a moderate, but statistically significant correlation between total PANSS and SQLS-R4 scores at baseline in the cariprazine group in both the ITT and PNS population which was not present in the placebo or aripiprazole groups in either population. After 6 weeks of treatment, improvements in PANSS total score were significantly associated with improvements in SQLS-R4 across all groups in the ITT population (*p* ≤ 0.001); this association was only present for patients treated with cariprazine in the PNS population. In the PNS population, a significant correlation was observed between CDR-POA and SQLS-R4 for patients treated with aripiprazole (*p* ≤ 0.01). Despite there being no significant correlation between PANSS-FSNS and SQLS-R4 baseline scores in any group, there was a highly significant correlation between the changes of these scores from baseline to week 6 that was specific to the cariprazine group in both ITT and PNS populations (*P* ≤ 0.0001).

**Table 3:** Correlations between baseline and change from baseline scores between PANSS, PANSS-FSNS, CDR-POA, CDR-COA and SQLS-R4 scores.

Pearson correlation coefficient (n)		Correlations in baseline scores				Correlations in change from baseline scores			
		PANSS- FSNS	CDR-POA	CDR-COA	SQLS-R4	PANSS- FSNS	CDR-POA	CDR-COA	SQLS-R4
ITT population									
Total PANSS	Placebo	0.543⁎⁎ (294)	0.003 (287)	−0.0254 (285)	−0.017 (292)	0.822⁎⁎ (184)	0.178 (176)	−0.188 (173)┼	0.253⁎ (182)
	CAR	0.560⁎⁎ (599)	0.007 (587)	−0.0114 (584)	0.144⁎ (591)	0.737⁎* (385)	0.102 (366)	−0.109 (359) ^┼^	0.369⁎⁎ (377)
	ARI	0.515⁎⁎ (150)	0.058 (149)	−0.0323 (149)	0.039 (150)	0.753⁎⁎ (115)	0.026 (106)	−0.111 (106)	0.321* (115)
PANSS-FSNS	Placebo		0.053 (287)	−0.0714 (285)	−0.056 (292)		0.134 (176)	−0.169 (173) ^┼^	0.168 (182)
	CAR		0.018 (587)	−0.0717 (584)	0.088 (591)		0.035 (366)	−0.107 (359) ^┼^	0.267⁎⁎ (377)
	ARI		0.111 (149)	−0.1528 (149)	−0.021 (150)		−0.093 (106)	−0.004 (106)	0.178 (115)
CDR-POA	Placebo			−0.724 (285)⁎*	0.017 (287)			−0.434 (173)⁎⁎	0.082 (175)
	CAR			−0.719 (584)⁎⁎	−0.039 (581)			−0.394 (359)⁎⁎	0.027 (362)
	ARI			−0.671 (149)⁎⁎	−0.160 (149)			−0.773 (107)⁎⁎	0.062 (107)
CDR-COA	PBO				−0.023 (285)				−0.002 (172)
	CAR				0.010 (578)				0.027 (355)
	ARI				0.130 (149)				0.015 (107)

PNS population									
Total PANSS	Placebo	0.459⁎⁎ (66)	0.072 (64)	−0.142 (64)	0.016 (65)	0.834⁎⁎ (43)	0.051 (41)	−0.050 (41)	0.388 (42)
	CAR	0.537⁎⁎ (105)	0.025 (105)	−0.056 (105)	0.304# (104)	0.817⁎⁎ (69)	0.066 (67)	−0.130 (67)	0.601⁎⁎ (69)
	ARI	0.581⁎⁎ (44)	−0.035 (44)	−0.093 (44)	0.114 (44)	0.837⁎⁎ (34)	0.126 (31)	−0.154 (31)	0.296 (34)
PANSS-FSNS	Placebo		0.243 (64)	−0.227 (64)	−0.002 (65)		0.093 (41)	−0.088 (41)	0.206 (42)
	CAR		−0.041 (105)	0.002 (105)	0.148 (104)		0.057 (67)	−0.154 (67)	0.470⁎⁎ (69)
	ARI		−0.072 (44)	0.035 (44)	−0.041 (44)		−0.158 (31)	−0.024 (31)	0.151 (34)
CDR-POA	Placebo			−0.702 (64)⁎⁎	0.307 (64)			−0.076 (41)	0.003 (41)
	CAR			−0.769 (105)⁎⁎	0.068 (104)			−0.545 (67)⁎⁎	0.060 (67)
	ARI			−0.710 (44)⁎⁎	−0.171 (44)			−0.750 (31)⁎⁎	0.488# (31)
CDR-COA	Placebo				−0.221 (64)				0.214 (41)
	CAR				−0.126 (104)				−0.116 (67)
	ARI				0.141 (44)				−0.241 (31)

Abbreviations: ARI, aripiprazole; CAR, cariprazine; CDR-PoA, Cognitive Drug Research - Power of Attention; ITT, intention to treat; PANSS, Positive and Negative Syndrome Scale; PANSS-FSNS, Positive and Negative Syndrome Scale factor score for negative symptoms; PANSS-FSPS, Positive and Negative Syndrome Scale factor score for positive symptoms; PNS, predominant negative symptoms; SQLS-R4, Schizophrenia Quality of Life Scale Revision 4.

⁎⁎ p ≤ 0.0001.

⁎ p ≤ 0.001.

# p ≤ 0.01.

┼ p ≤ 0.05.

## 4 Discussion

In the ITT population, the robust effect of cariprazine on the PANSS-FSNS is consistent with previous findings (e.g., Earley et al., 2019; Németh et al., 2017) suggesting a particular benefit of this agent on negative symptoms. In this analysis, aripiprazole was not associated with a significant benefit on PANSS-FSNS in the PNS subgroup, suggesting this may not be a class effect of the partial agonists, but may be specific to cariprazine. These data suggest pragmatic optimism for improved outcomes with individualized pharmacotherapy for patients with PNS.

These post-hoc analyses were undertaken to further explore treatment outcomes with cariprazine on QoL and attention measures in relation to negative symptomology in patients with acute exacerbation of schizophrenia. Outcomes using the pooled ITT data from two placebo-controlled trials of cariprazine showed positive changes in measures of QoL and attention with short-term (6-weeks) treatment; particularly among the subgroup of patients presenting with PNS. The *post-hoc* nature of these analyses do not result in definitive conclusions, but do provide incentive for subsequent, prospective exploration of potential connections between changes in measures of QoL and cognition (e.g., attention) in relation to improvements in negative symptoms. Such an exploration should ensure balanced baseline scores in measures of attention and global cognition, the absence of which are a limitation to the current conclusions.

Primary negative symptoms are intrinsic to the disorder of schizophrenia, whereas secondary negative symptoms may be caused by other factors such as poorly controlled positive symptoms, comorbid depression, medication side-effects, social deprivation or related to substance use; secondary negative symptoms typically improve with treatment of the underlying cause (Kirschner et al., 2017; Kirkpatrick, 2014). As detailed above, both this analysis and the head-to-head study of cariprazine vs. risperidone (Németh et al., 2017) used the terminology “predominant” negative symptoms, recognizing some negative symptoms (and changes to those symptoms) may have been secondary to other causes. As additional areas for future exploration outside of post-hoc analysis, it would be beneficial to better control for primary vs. secondary negative symptoms and more directly examine, via comparative methodologies, whether dopamine D_2_ partial agonist medications have a lower liability to induce secondary negative symptoms or if the impact on negative symptoms (either primary or secondary) is more closely attributed to the totality of a molecule's receptor binding profile and proposed mechanism of action (i.e., D_3_, 5HT, and other receptor effects).

The interplay between changes in negative symptoms, QoL, attention, and treatment remains difficult to interpret. The findings of the present analysis suggest cariprazine has a beneficial effect on attention even within the 6-week duration. While a placebo response was seen in PANSS measures and in the assessment of QoL, this was not the case with respect to measures of attention; the placebo groups consistently demonstrated deteriorations over the 6-week duration of the studies. This suggests that for patients with schizophrenia, active pharmacological treatment may be required to maintain or improve measures of attention. Correlation analysis was undertaken to further examine the relationships between symptom scores, attention scores, and QoL. No correlation was detected between post-treatment attention scores with changes in either total PANSS or in PANSS-FSNS scores. Correlations between QoL improvements and PANSS scores were more complex. Improvement in total PANSS was correlated with improvements in QoL among all groups in the ITT population, but this correlation was only apparent for the cariprazine group in the PNS population. This may reflect the effect of cariprazine on negative symptoms given the degree of correlation between total PANSS and QoL scores was higher in the cariprazine-treated PNS population compared to the ITT population, and that the correlation between PANSS-FSNS scores and QoL improvements was only apparent in the cariprazine group in both the ITT and PNS populations. Interestingly, there was a correlation between attention and QoL scores in only the aripiprazole-treated patients in the PNS population. These associations should be further explored in a larger sample size to provide greater statistical power.

An alternative hypothetical interpretation is that improvements in negative symptoms and psychosocial functioning in favor of cariprazine over risperidone (Németh et al., 2017) were partially driven by favorable changes in attention among patients with PNS. Whether this effect is specific to cariprazine, potentially because of its unique D_3_-preferring activity, or more related to duration of treatment, is not known based on current evidence.

The assessment of QoL outcomes could also benefit from a study of longer duration. In the current analysis, the PNS populations for both aripiprazole and cariprazine demonstrated results similar to the ITT population in both magnitude of SQLS-R4 score change and significance of results. This could reflect the short duration of the studies (6 weeks), and improvements in a wide range of symptoms (including positive symptoms) given that patients were experiencing acute exacerbation at study entry. Meaningful, comprehensive changes on QoL, if related to negative and/or cognitive symptoms, would likely take longer to see fully; as demonstrated by the between-group differences on the PANSS-FSNS seen in the 26-week, head-to-head study of risperidone vs. cariprazine (Németh et al., 2017). A longer-term study designed to specifically investigate QoL questions in both a general population of individuals with schizophrenia, and those with PNS, is a potential area of future research.

These analyses show several differences between the completer and last visit data, with the completer results showing more robust treatment effects across most measures. This supports the well-established mantra of pharmacologic therapy: treatments are most effective when taken as prescribed and may be particularly true among patients with prominent negative symptoms. Furthermore, these findings highlight the desirability of including completer analyses in the evaluation of medical interventions, as it may be more feasible to test hypotheses of interest and/or subpopulations among adherent completers than in an ITT analysis.

The study involving both cariprazine and aripiprazole was not designed to compare the two active agents; aripiprazole was included as an active-control arm for assay sensitivity. Possible differences between cariprazine and aripiprazole suggested by these analyses are not conclusive and the relative sample sizes of aripiprazole for these analyses are relatively small. While acknowledging these limitations, the possibility that there may be clinically important differences cannot be ruled out. While both active therapies significantly improved QoL, the magnitude was numerically greater with aripiprazole. With respect to the findings on attention measures, the D_3_-preferring activity of cariprazine may be an important distinguishing characteristic. Dopamine D_3__−_receptor effects may be particularly important in the dorsolateral prefrontal cortex, where too little dopamine may drive negative and cognitive symptoms and where a rebalancing of dopamine may be important to reduce negative symptoms and perhaps cognitive symptoms (Frankle et al., 2022; Fuentes-Claramonte et al., 2022; Smucny et al., 2022; Ott and Nieder, 2019; Rao et al., 2019).

## 5 Conclusions

In conclusion, this post-hoc analysis of two placebo-controlled studies investigating cariprazine for the treatment of acute exacerbations of schizophrenia suggests that this agent is associated with potentially beneficial effects on measures of attention and QoL, and these effects may be more pronounced among individuals with schizophrenia with PNS.

## CRediT authorship contribution statement

**Oloruntoba J. Oluboka:** Writing – review & editing, Writing – original draft, Supervision, Methodology, Conceptualization. **Andrea Bardell:** Writing – review & editing, Methodology, Conceptualization. **Howard C. Margolese:** Writing – review & editing, Methodology, Conceptualization. **Philip G. Tibbo:** Writing – review & editing, Methodology, Conceptualization. **Lisa Buchy:** Writing – review & editing, Methodology, Conceptualization. **Christine Di Cresce:** Writing – review & editing, Writing – original draft, Project administration, Methodology, Funding acquisition, Conceptualization. **Jun Yu:** Writing – review & editing, Methodology, Formal analysis, Data curation. **Roger S. McIntyre:** Writing – review & editing, Methodology, Conceptualization.

## Ethics statement

The studies were approved by an Institutional Review Board (United States) or ethics committee (non–US sites). ICH-E6 Good Clinical Practice guidelines were followed, and written informed consent was obtained from all participants.

## Funding sources

Original research relating to this manuscript was funded by 10.13039/100005632Forest Laboratories LLC, an 10.13039/100007819Allergan affiliate (Jersey City, New Jersey) and 10.13039/501100003358Gedeon Richter Plc (Budapest Hungary).

## Declaration of competing interest

OJO has received honoraria for his participation as a consultant, advisory committee member and/or as a speaker at educational events for Allergan, CPD Network, HLS, Janssen, Lundbeck, Otsuka, Pfizer and Sunovion. He has also received grants / research support from Lundbeck-Otsuka Alliance.

AB has received honoraria for her participation as a consultant, advisory committee member and/or as a speaker at educational events for AbbVie, GSK, HLS Therapeutics, Janssen, Lundbeck and Otsuka. She has also received grants / research support from-10.13039/100009155Otsuka.

HCM has received honoraria for his participation as a consultant, advisory committee member and/or as a speaker at educational events for AbbVie, HLS Therapeutics Inc., Janssen, Lundbeck, Otsuka, Sunovion and Teva. He has also received grants / research support from 10.13039/100005294MGH Hospital Foundation, SyneuRX and AiFred.

PT has received honoraria for his participation in advisory boards and/or as a speaker from Janssen, Otsuka Lundbeck and AbbVie. He has received investigator initiated research grant support from Janssen.

CdC and LB are employees of AbbVie Corporation (Canada).

JY is an employee of AbbVie Corporation (USA).

RSM has received research grant support from CIHR/GACD/National Natural Science Foundation of China (NSFC) and the Milken Institute; speaker/consultation fees from Lundbeck, Janssen, Alkermes,Neumora Therapeutics, Boehringer Ingelheim, Sage, Biogen, Mitsubishi Tanabe, Purdue, Pfizer, Otsuka, Takeda, Neurocrine, Sunovion, Bausch Health, Axsome, Novo Nordisk, Kris, Sanofi, Eisai, Intra-Cellular, NewBridge Pharmaceuticals, Viatris, AbbVie, and Atai Life Sciences. RSM is CEO of Braxia Scientific Corp.

## Appendix A Supplementary data

Supplementary material Image 1
