# Supplementary material for: Effect of cariprazine on attention and quality of life in patients with predominant negative symptoms of schizophrenia: A post-hoc analysis
Source: Schizophr Res Cogn. 2025 Mar 8;40:100355. doi: 10.1016/j.scog.2025.100355 (PMC11930433; doi:10.1016/j.scog.2025.100355)

**Effect of Cariprazine on Attention and Quality of Life in Patients With Predominant Negative Symptoms of Schizophrenia: A Post-hoc Analysis**

**Supplemental Materials**

Table S1. **Proportion of Subjects Achieving a Total PANSS Response of 50% or Greater at Last Visit and Week 6**

**2A. Cariprazine *vs*. Placebo**

|  | **ITT (n=905)** | | **PNS (n=177)** | |
| --- | --- | --- | --- | --- |
| **% achieving ≥50% response (n/N)** | **CAR** | **PBO** | **CAR** | **PBO** |
| Last visit | 3.80% (23/606) | 2.34% (7/299) | 4.55%  (5/110) | 0%  (0/67) |
| Week 6 | 5.38%  (21/390) | 3.74%  (7/187) | 5.33%  (4/75) | 0%  (0/45) |

**2B. Aripiprazole *vs*. Placebo**

|  | **ITT (n=449)** | | **PNS (n=109)** | |
| --- | --- | --- | --- | --- |
| **% achieving ≥50% response (n/N)** | **ARI** | **PBO** | **ARI** | **PBO** |
| Last visit | 2.00% (3/150) | 2.34% (7/299) | 4.76%  (2/42) | 0%  (0/67) |
| Week 6 | 2.61%  (3/115) | 3.74%  (7/187) | 6.25%  (2/32) | 0%  (0/45) |

Abbreviations: ARI, aripiprazole; CAR, cariprazine; ITT, intention to treat; PANSS, Positive and Negative Syndrome Scale; PBO, placebo; PNS, predominant negative symptoms

**Supplemental Figures**

Figure S1. Least-square mean changes (LSMC) (± Standard Error of the Mean [SEM]) and least-square mean differences (LSMD) in The Schizophrenia Quality of Life Scale Revision 4 (SQLS-R4) score vs. Placebo (Completers) in the intention-to-treat (ITT) and predominant negative symptom (PNS) populations at week 6 for the last visit analyses.

S1A. Cariprazine (CAR) vs. Placebo (PBO)


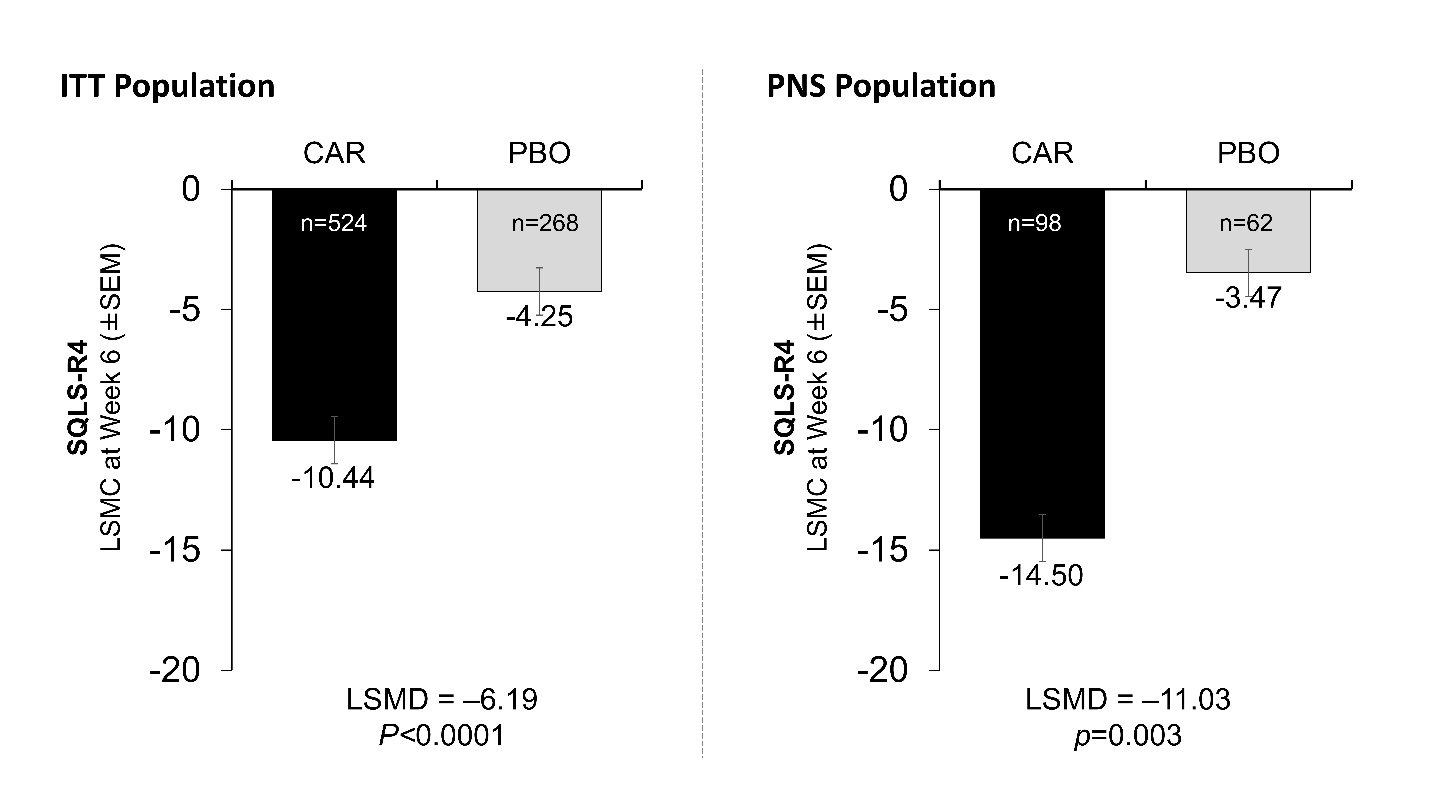


S1B. Aripiprazole (ARI) *vs*. Placebo (PBO)


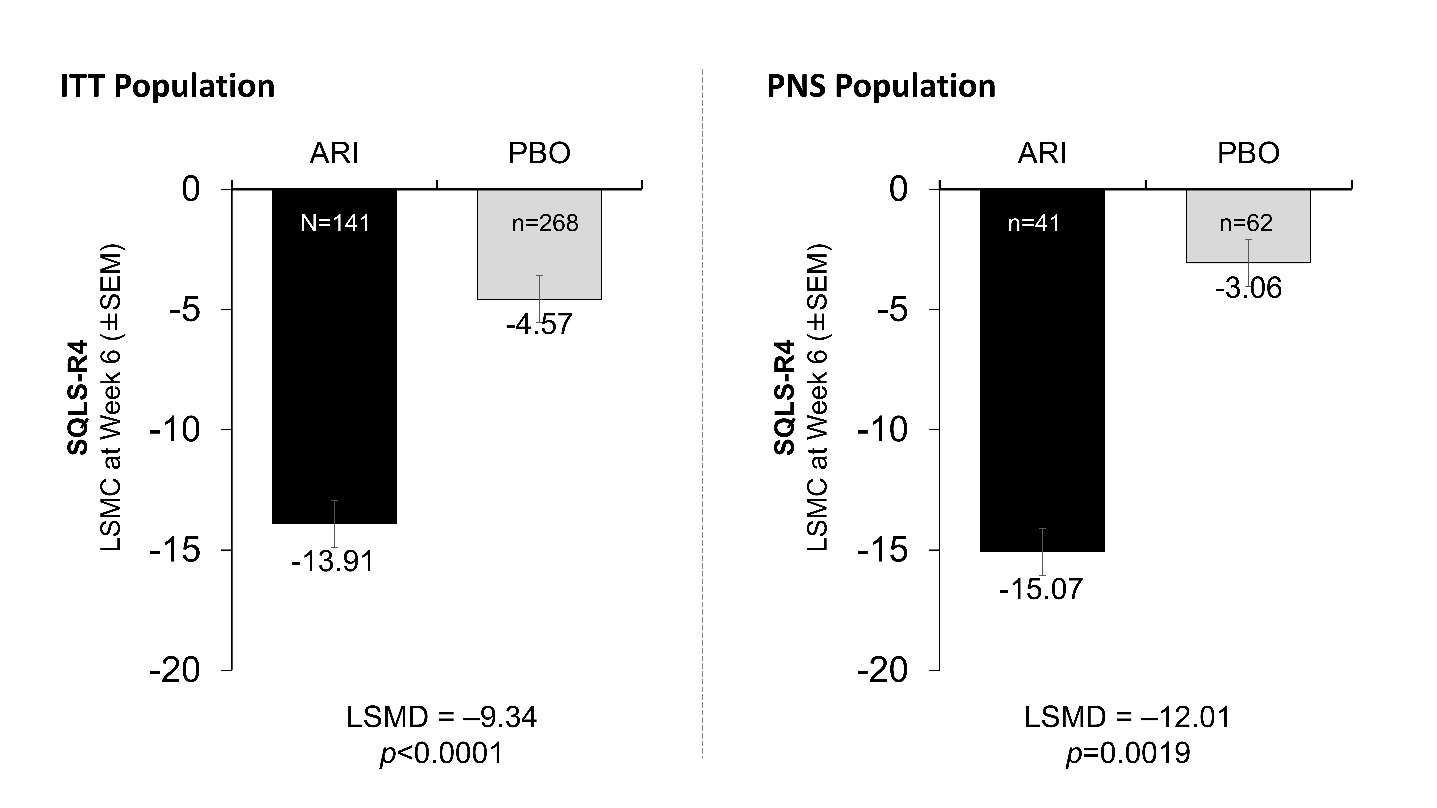


Figure S2. Least-square mean changes (LSMC) (± Standard Error of the Mean [SEM]) and least-square mean differences (LSMD) in Focused Attention based on the Cognitive Drug Research - Power of Attention (CDR-PoA) score vs. Placebo (Completers) in the intention-to-treat (ITT) and predominant negative symptom (PNS) populations at week 6 for the last visit analyses.

S2A. Cariprazine (CAR) vs. Placebo (PBO)


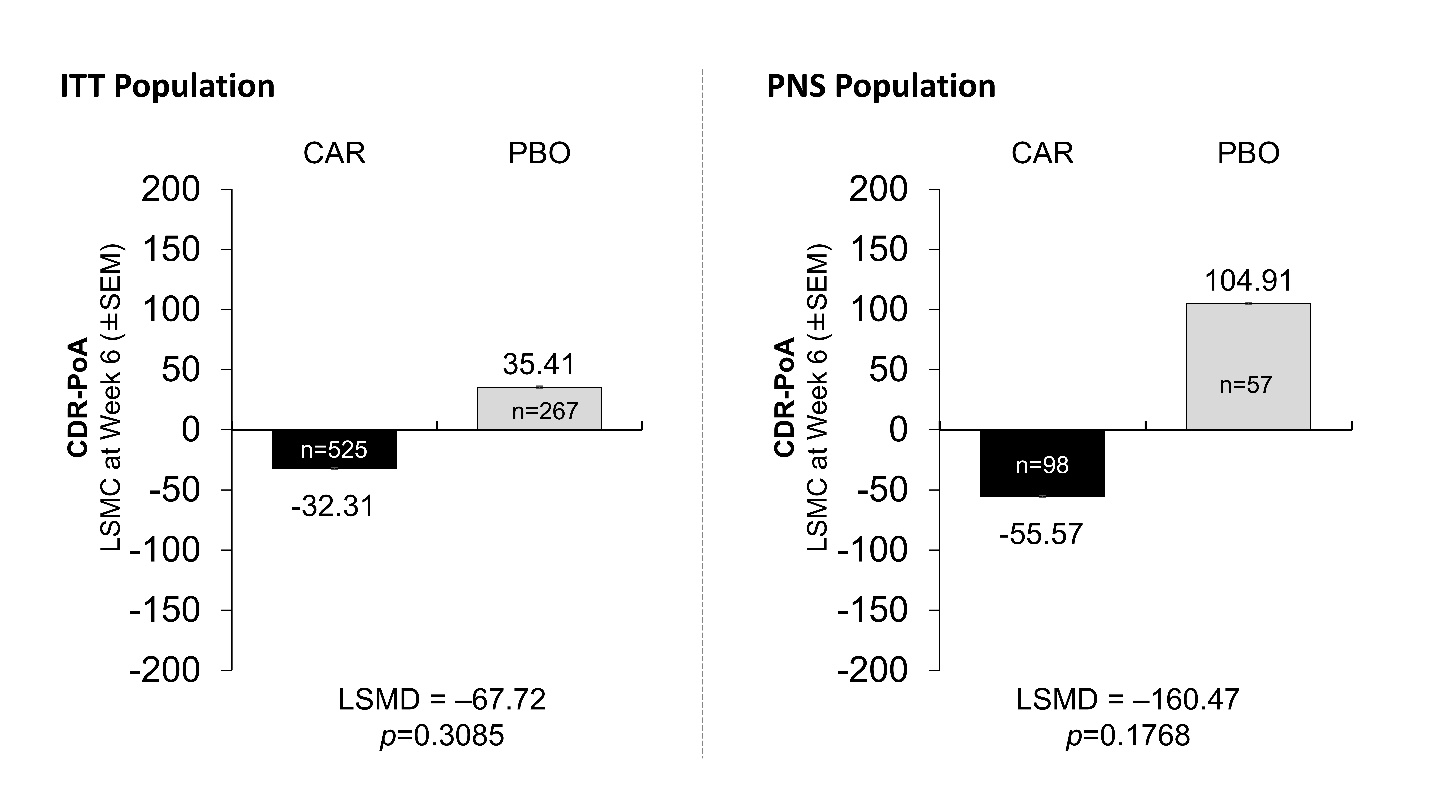


S2B. Aripiprazole (ARI) *vs*. Placebo (PBO)


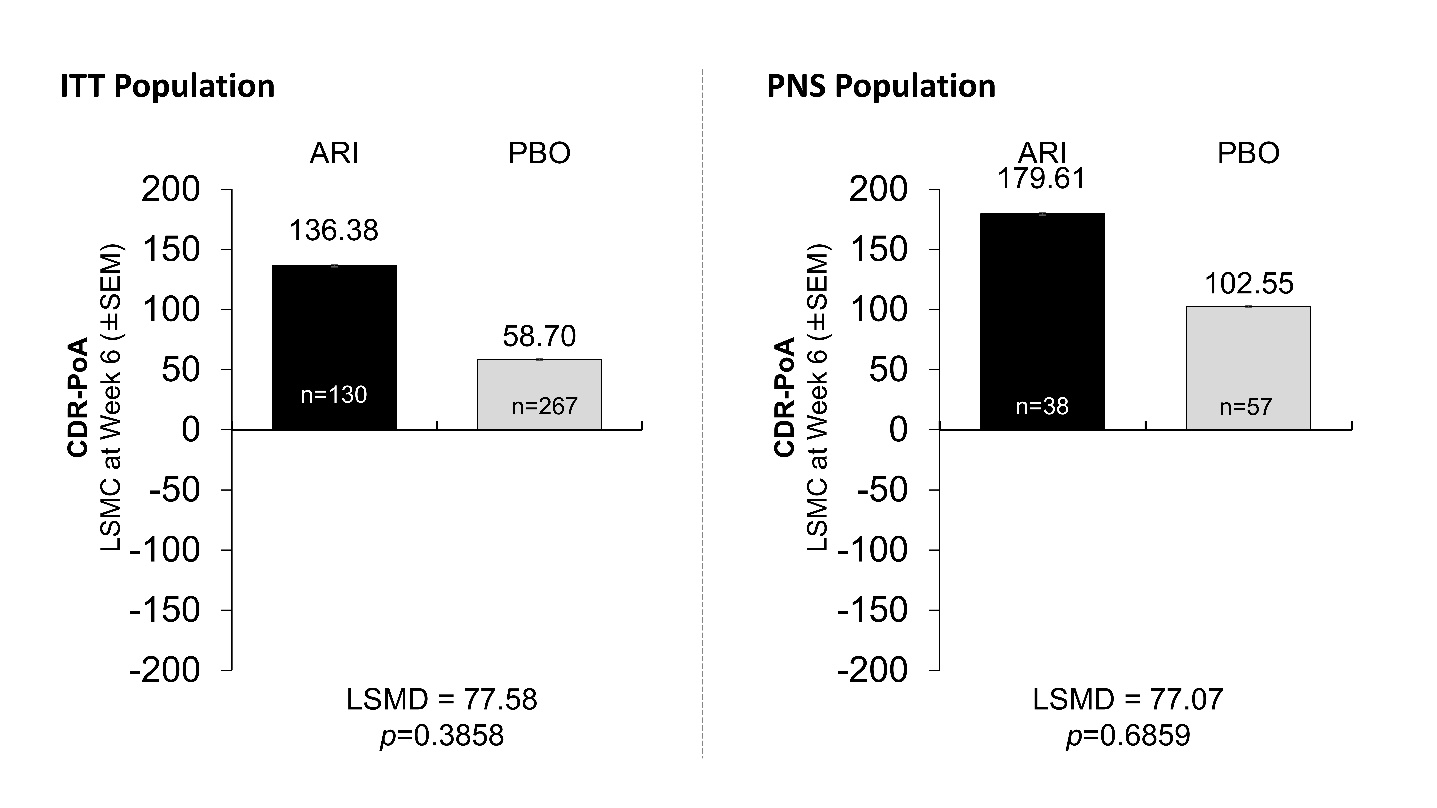


Figure S3. Least-square mean changes (LSMC) (± Standard Error of the Mean [SEM]) and least-square mean differences (LSMD) in Sustained Attention based on the Cognitive Drug Research - Continuity of Attention (CDR-CoA) score vs. Placebo (Completers) in the intention-to-treat (ITT) and predominant negative symptom (PNS) populations at week 6 for the last visit analyses.

S3A. Cariprazine (CAR) vs. Placebo (PBO)


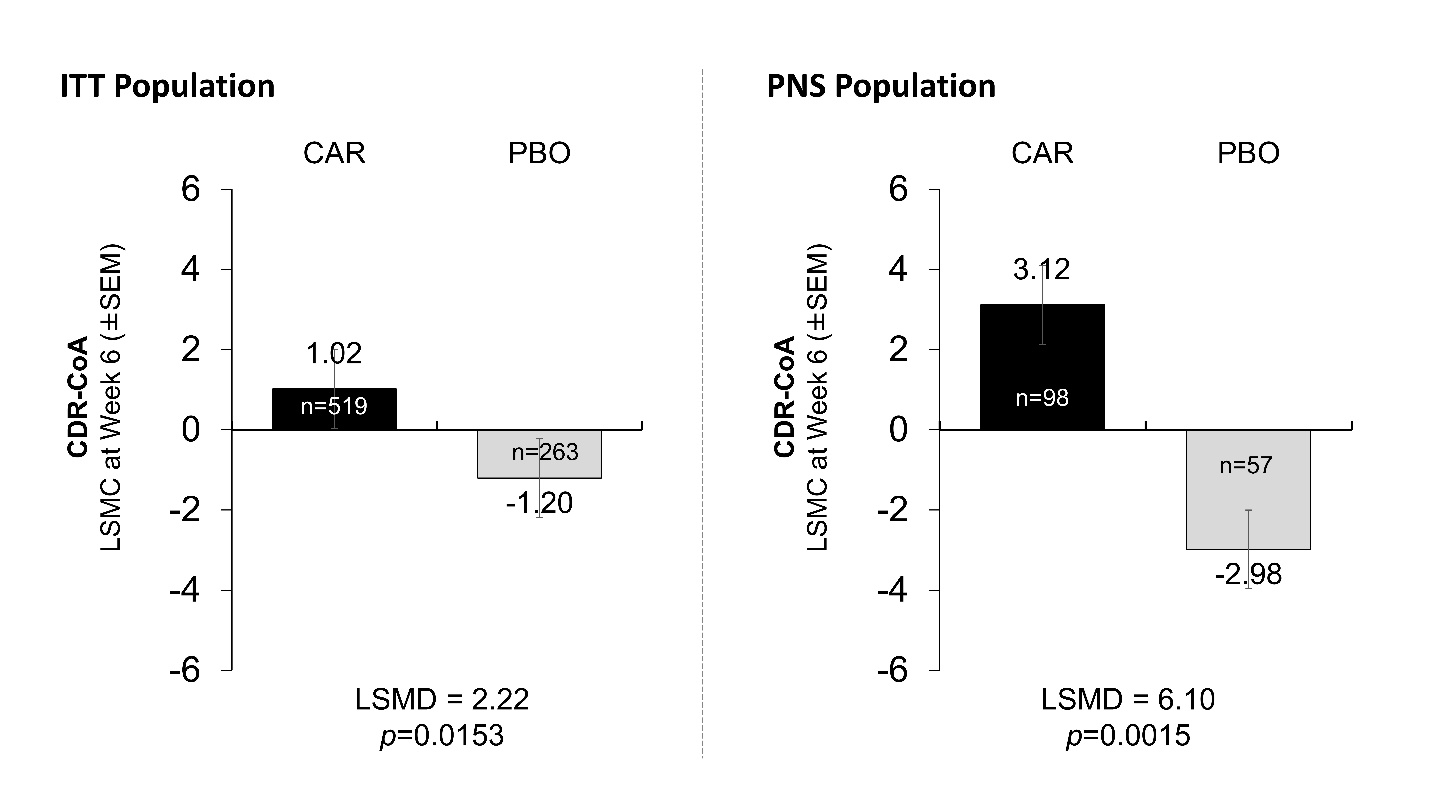


S3B. Aripiprazole (ARI) *vs*. Placebo (PBO)


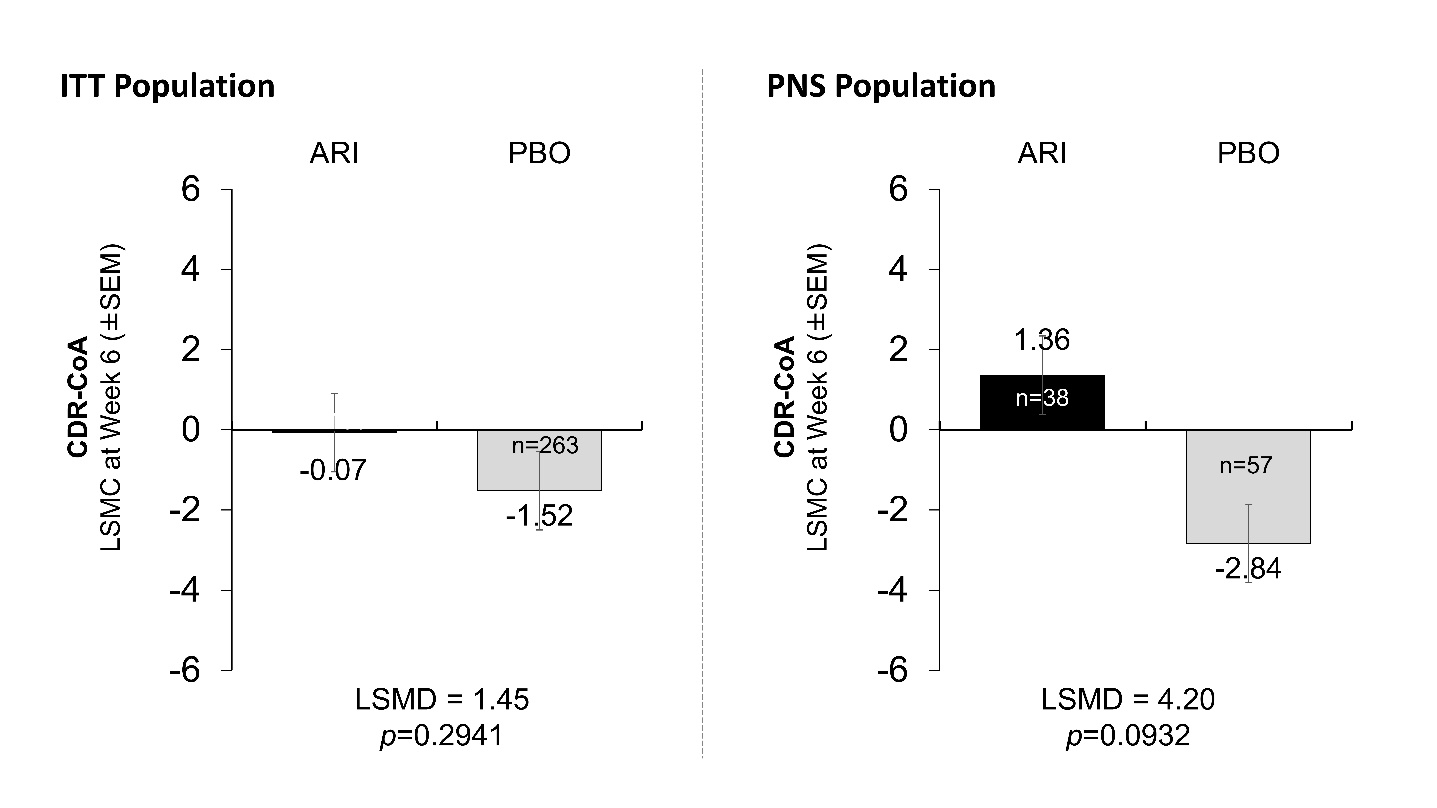

Supplement: Supplementary file 1 — Supplementary material [file mmc1.docx]
